# Oral Bisphosphonate Therapy for Osteogenesis Imperfecta: A Systematic Review and Meta‐Analysis of Six Randomized Placebo‐Controlled Trials

**DOI:** 10.1111/os.12611

**Published:** 2020-06-26

**Authors:** Zhi‐min Ying, Bin Hu, Shi‐gui Yan

**Affiliations:** ^1^ Department of Orthopaedic Surgery, the Second Affiliated Hospital School of Medicine, Zhejiang University Zhejiang China

**Keywords:** Alendronate, Clodronate, Etidronate, Oral bisphosphonates, Esteogenesis imperfecta

## Abstract

**Objective:**

To assess the effectiveness and safety of oral bisphosphonates in increasing bone mineral density (BMD), reducing fractures, and improving clinical function in patients with osteogenesis imperfecta (OI).

**Methods:**

Studies were eligible for inclusion if they were randomized controlled trials of directly comparing oral bisphosphonate therapy with placebo‐group in OI patients. Data synthesis regarding to bone mineral density as measured by dual‐energy X‐ray absorptiometry (DEXA), decreased fracture incidence, change in biochemical markers of bone and mineral metabolism, bone histology, growth, bone pain, quality of life, and others were assessed, and meta‐analysis done when possible.

**Results:**

From 98 potential references and six randomized controlled studies a total of 263 participants receiving oral bisphosphonates and 143 placebo treatments contributed data to meta‐analysis. Pooled meta‐analysis of three studies suggested that there was significant difference between bisphosphonate treated group and placebo in number of patients with at least one fracture (mean difference 0.53, 95% confidence interval 0.32–0.89, *P* = 0.02). Pooled meta‐analysis of two studies suggested that significant difference was noted between bisphosphonate treated group and placebo in mean percentage change in spine BMD (T‐score) (mean difference 28.43, 95% confidence interval 7.09‐49.77, *P* = 0.009). The similar effect was shown in the term of mean change (Z‐score) in spine BMD.

**Conclusions:**

Significant improvement in lumbar areal BMD in patients affected with OI has been shown when treated with oral bisphosphonates, even though only a small population was enrolled. We cannot draw a definite conclusion that the increase in BMD can be translated into fracture reduction and clinical functional improvement. The optimal method, dose, type, initiation, and duration of oral bisphosphonates therapy still remains unclear. Well‐designed, adequately‐powered, placebo‐controlled RCTs investigating the effects of oral bisphosphonates on fractures reduction and improvement in quality of life in both children and adults are studied here.

## Introduction

Osteogenesis imperfecta (OI) is an inherited disorder of connective tissue, caused by mutations in the genes that encode type I collagen^1^, ^2^, ^3^. OI is characterized by increased bone fragility of varying severity and low bone mass^4^, ^5^. It commonly presents with joint hypermobility, blue or grey‐blue scleral color, dentinogenesis imperfecta, and premature hearing loss. This genetic rare disease is almost always caused by mutations in one of the two genes encoded with type I αchains (*COL1A1* and *COL1A2)* 6, 7. The incidence of osteogenesis imperfecta is estimated to be between one and two per 20000 people^8^, ^9^, ^10^. Normal bone matrix is composed of 10% non‐collagenous proteins and 90% type I collagen fibers which provide bone resilience. Individuals with OI have less and/or poorer quality type I collagen than unaffected people, causing their bones to deform and/or fracture. Further refinement of these classifications was made with molecular genetic analyses^3^. Recently, the addition of OI types V, VI and VII have been proposed^9^. It is unclear whether OI types V, VI and VII will be classified with OI in the future as these individuals do not have evidence of type I collagen mutations.

Intravenous and oral bisphosphonates are first‐line established therapy for the treatment of most patients with osteoporosis, with proven efficacy to reduce fracture risk at the spine, hip, and other nonvertebral skeletal sites^11^. Bisphosphonates act by inactivating osteoclasts, the cells that break down bone tissue, thereby inhibiting bone resorption^43^. There are two different types of bisphosphonates, nitrogenous and non‐nitrogenous. Nitrogenous bisphosphonates including alendronate, eridronate, olpadronate, pamidronate, risedronate and zolendronate disrupt osteoclast formation, survival and cytoskeletal dynamics. Non‐nitrogenous bisphosphonates including clodronate and etidronate and tiludronate initiate osteoclast apoptosis. The bisphosphonates vary in their efficacy and absorption when taken orally, making direct comparison challenging. They increase the areal bone mineral density (BMD) and reduce the incidence of osteogenesis imperfecta. Beneficial effects have also been reported in children with osteogenesis imperfecta^12^, ^13^, ^14^, ^15^, ^16^, ^17^, ^18^, ^29^.

Several important quasi‐randomized or randomized controlled trials presented increase of BMD and decrease of reported fracture rates in children or adults with osteogenesis imperfecta by cyclic intravenous neridronate and pamidronate^19^, ^20^, ^21^. But treatment with intravenous bisphosphonate needs medication at regular intervals at home or during hospital stays. This invasive and inconvenient medication administration unavoidably affects schooling, distracting parents from their work commitments. The intravenous administration is also unfriendly to the patients especially to the children. Oral treatment provides merits in terms of convenience, cost, and reduced individual distress. Therefore, there is still no definite conclusion as to whether oral administration of bisphosphonate can improve quality of life in adults and children with osteogenesis imperfecta, especially regarding the reduced fracture rate.

The purpose of this study was to conduct a systematic review and meta‐analysis of recent studies to address if oral bisphosphonate therapy for osteogenesis imperfecta was equivalent to the placebo group regarding bone mineral density as measured by dual‐energy X‐ray absorptiometry (DEXA), decreased fracture incidence, lessening of deformity, reduced pain, and improved growth and mobility. Our hypothesis was that the oral bisphosphonate therapy would offer the patients with improved quality of life and reduced fracture rate than placebo group.

## Methods

### *Inclusion and Exclusion Criteria*

Studies were eligible for inclusion if they were randomized controlled trials that directly compared oral bisphosphonate therapy with placebo‐group in OI patients, and measured primary outcomes of bone mineral density as measured by dual‐energy X‐ray absorptiometry (DEXA), decreased fracture incidence, lessening of deformity, reduced pain, and improved growth and mobility (Table 1). These variables were selected because at least half of the studies included each of these measures. Children (defined as age 0 to 18 years) and adults with OI diagnosed using accepted diagnostic criteria, based on clinical or laboratory findings, or both, were eligible. Individuals affected with all types of OI are included in this review. We excluded randomized controlled trials in which any enrolled patients received the bisphosphonate therapy using IV administration. Although pathology features harvested from the operated site would be the ideal outcome measure, this would require following large number of participants for decades. No such studies have been done, so we used the various Clinical Scores System and radiologic results as surrogate outcomes, as are commonly used in patients. Clinical Scores System and radiologic results have been associated with clinical effects.

**Table 1:** PICOS criteria for inclusion and exclusion of studies

Parameter	Inclusion criteria	Exclusion criteria
Patients	Children (defifined as age 0 to 18 years) and adults with OI diagnosed using accepted diagnostic criteria, based on clinical or laboratory findings, or both. Individuals affected with all types of OI are included in this review.	Randomized controlled trials in which any enrolled patients who receive the bisphosphonate therapy using IV administration
Intervention	Oral Bisphosphonate	
Comparator	Placebo	
Outcomes	Bone mineral density as measured by dual‐energy X‐ray absorptiometry (DEXA), decreased fracture incidence, change in biochemical markers of bone and mineral metabolism, bone histology, growth, bone pain, quality of life and others were assessed	Studies without defined clinical outcomes
Study design	Randomized controlled trials	Non‐randomized controlled trials; Retrospective, prospective, or concurrent cohort studies; Cross sectional studies

### *Searching Strategy and Selection Method*

Review protocol described by Spindle *et al*. and Wright *et al*.^23^, ^24^ were employed. The electronic literature search was last updated on 28 August 2019. Without language restrictions, we searched the Medline (1966‐present), Cochrane Central Register of Controlled Trials, and EMBASE (1980‐present), CINAHL (1982 to present), AMED (1985 to present), and ISI Web of Science (1945 to present) for the terms “oral bisphosphonate”, “alendronate”, “clodronate”, “etidronate”, “ibandronate”, “olpadronate”, “risendronate”, “tiludronate”, and “osteogenesis imperfecta”. The nine terms were searched individually and were combined with Boolean terms. No exclusions such as publication year or journal name were specified in the search strategy. We manually searched conference abstract issues of key journals for 2000–2019: *European Journal of Pediatrics*, *Journal of Pediatric Orthopaedics*, *The Journal of Bone and Joint Surgery* (Am or Br version), *The Journal of Clinical Endocrinology and Metabolism*, *Clinical Orthopaedics and Related Research*, and the *Journal of Bone and Mineral Research*. We examined the reference lists and ISI citations of all included studies. Two reviewers assessed potentially relevant articles against the inclusion criteria. Inclusion criteria included English‐language Level I of evidence studies involving directly comparing the effects of oral bisphosphonates in all types of OI. Exclusion criteria included non‐randomised control studies, intravenous bisphosphonate, and follow‐up of the study population less than 70%. PubMed searches from 1966 to 2019 using MESH terms, “osteogenesis imperfecta”, “randomized controlled trial”, and “Randomised Controlled Trials,” were also carried out by the authors.

### *Data Collection*

Two reviewers (Zhi‐min Ying, Bin Hu) independently reviewed each abstract and extracted data. The references for each of these studies also were examined for other relevant studies. If there were any discrepancies, a third individual reviewed the articles. We extracted change between the two different therapies in bone mineral density as measured by dual‐energy X‐ray absorptiometry (DEXA), decreased fracture incidence, change in biochemical markers of bone and mineral metabolism, bone histology, growth, bone pain, quality of life. Two reviewers independently assessed each trial's risk of bias, assessing factors such as randomization, allocation concealment, blinding, completeness of outcome assessment, and selective reporting. Where necessary we contacted authors to obtain information on primary outcome factors.

### *Assessment of Risk of Bias in Included Studies*

We assessed the methodological quality of the trials based on the method described by Jüni^25^. In addition, we described the generation of allocation sequence and concealment of allocation sequence as adequate, inadequate, or unclear. We also evaluated each trial for the degree of blinding and whether an intention‐to‐treat analysis was undertaken. The methodological quality ratings, and details as to why they assigned these ratings for each criterion was presented (Table 2). The authors have presented methodological quality ratings, and details as to why they assigned these ratings for each criterion. However, studies were not weighted on the basis of their assigned methodologic quality.

**Table 2:** Methodological quality of included studies

Study ID	Allocation concealment	Randomization	Blinding	Type of analysis
Sakkers 2004 (Olpadronate)	Responsibility of a trial management department	Randomization was by computer‐generated random numbers.	Stated that researchers Were blinded to treatment allocation.	Intention‐to‐treat.
2006 Chevrel (Alendronate)	Researchers responsible for seeing participants allocated the next available number on entry into the trial.	Randomization was computer‐generated.	Double‐blinded (study personnel and participants), using a matched placebo	Intention‐to‐treat.
Kok 2007 (Olpadronate)	Method not stated	Randomisation was performed using a list of computer generated random numbers to allocate patients to receive oral Olpadronate or placebo	Both Olpadronate and placebo were prepared as entericcoated tablets	Intention‐to‐treat.
Rauch 2009 (Risedronate)	Method not stated	Randomization by equal number to receive the same treatment	Not stated	Intention‐to‐treat.
Ward 2011 (Alendronate)	Method not stated	Patients were randomized in a 3:1 ALN to placebo ratio and stratified according to their weight at baseline to receive either ALN 5mg daily (those 40 kg) or ALN 10 mg daily (those 40 kg), or matching placebo.	The study was coordinated and organized under the control of an independent steering committee, whose members were not involved in the study as investigators.	Intention‐to‐treat.
Bishop 2013 (Risedronate)	Method not stated	Patients were stratified by age (4–9, 10–15 years) and randomly assigned to receive treatment for 1 year with risedronate tablets or placebo in a 2:1 ratio by a telephone‐based interactive voice response system in several permuted blocks of ten to 12	The study treatment was masked from patients, investigators, and study centre personnel during the first year. After the first year, all patients were given risedronate (open‐label phase)	intention‐to‐treat

### *Data Analysis*

We converted all the different outcomes to standardized mean differences, calculating a standardized mean difference of hanged from base‐line in treatment and control groups. Where clinically useful, we estimated a benefit in units of percentage change since baseline from the standardized mean differences by estimating the pooled standard deviation from the means of the standard deviation of the outcomes in double‐row and single‐row groups for each study, and multiplying the standardized mean differences by this.

We calculated statistical heterogeneity using a *χ* ^2^ test on N‐1 degrees of freedom, with significance conservatively set at 0.10. We also assessed inconsistency I^2^ using the formula [(Q‐df)/Q] × 100%, where Q is the *χ* ^2^ statistic and df is its degrees of freedom, to describe the percentage of the variability in effect estimates due to heterogeneity. We considered a value greater than 50% as denoting substantial heterogeneity. For each study, relative risks (RRs) with 95% confidence intervals (CIs) and standardized or weighted mean differences with 95% CIs were calculated for dichotomous outcomes and continuous outcomes, respectively.

A fixed‐effects or random‐effects model was applied dependent on the heterogeneity of the studies. Quality appraisal was performed according to the CONSORT 2010 checklist and Cochrane scale was used to assess the risk of bias^26^. When heterogeneity was considered substantial, we explored its causes by carrying out pre‐specified subgroup analyses where data were available; that is, subgroup by sex, tear size, base profiles, compliance, and adequacy of allocation concealment. Where there weren't clear clinical reasons or study methodology reasons for substantial heterogeneity between studies, we proceeded to meta‐analysis using random effects models. All analyses were carried out in Review Manager 5 (Computer program. Version 5.3. Copenhagen: The Nordic Cochrane Centre, The Cochrane Collaboration, 2014.). When possible we used intention to treat data in analyses, but if these were not available we used, in order of preference, data from available data or per protocol analyses. Assessment of publication bias was by funnel plot.

## Results

### *Description of Studies*

**Figure 1:**
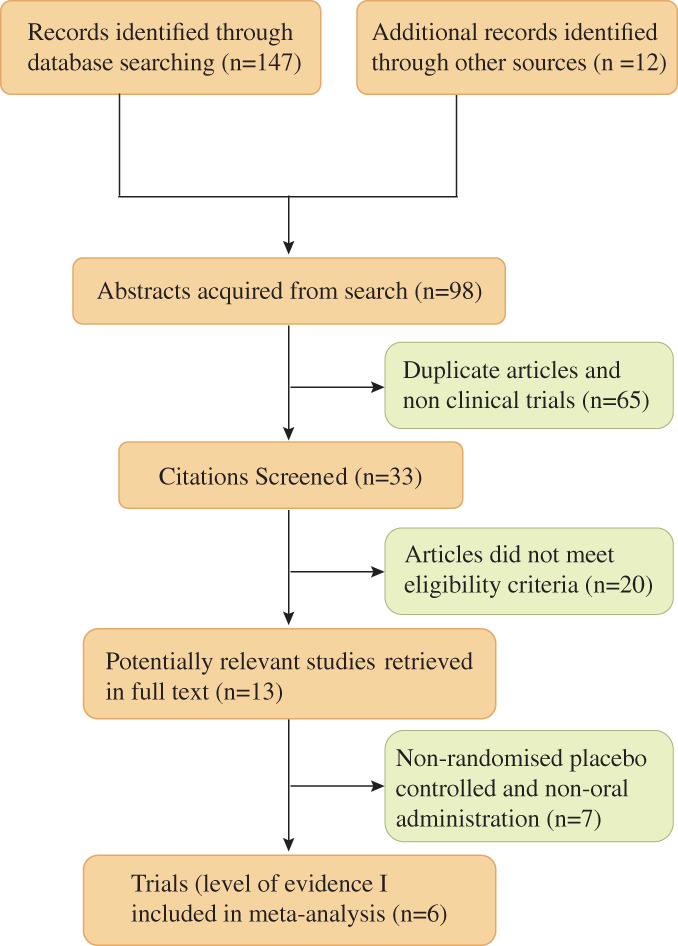
PRISMA Flow diagram of the inclusion process for the studies in the meta‐analysis.

Of the 13 studies were identified and reviewed, six randomized placebo‐controlled trials met the inclusion criteria and were included in this comparison^22^, ^27^, ^28^, ^29^, ^30^, ^31^ (see Fig. 1). Of these six randomized controlled studies, a total of 263 participants receiving oral bisphosphonates and 143 placebo treatments contributed data to meta‐analysis. Further details of the characteristics of included studies can be obtained in Tables 3 and 4. Trials were excluded if they are not RCTs or they did not evaluate effect of medicine bisphosphonate on OI patients or they did not assess the clinical outcome as in the form of bone density or fracture reduction. Five trials are investigating the effect of oral bisphosphonate clinical results in adults with osteogenesis imperfecta while only one trial studied the effect of oral bisphosphonate in children with osteogenesis imperfecta. They all have two studies that evaluated alendronate, risedronate and olpadronate separately.

**Table 3:** Demographics of clinical studies included in meta‐analysis

Study ID	Intervensions	Number of patients	Mean age (SD)	Women/Male	Osteogenesis Imperfecta(OI) Type	Duration
Sakkers 2004^27^	Olpadronate	16	10.0 (3.1)	7/9	Type I 4, Type III 4， Type IV 8	2 years
	Placebo	18	10.7 (3.9)	11/7	Type I 9, Type III 5， Type IV 4	
Chevrel 2006^28^	Alendronate	31	36 (12)	15/33	Type I 29， Type IV 2	3 years
	Placebo	33	37 (12)	10/20	Type I 33， Type IV 0	
Kok 2007^22^	Olpadronate Placebo	16 18	10.0 (3.1) 10.7 (3.9)	7/9 11/7	Type I 4， Type III 4， Type IV 8， Type I 9， Type III 5， Type IV 4	2 years
Rauch 2009^29^	Risedronate	13	11.7 (3.6)	5/8	Type I 13	2 years
	Placebo	13	11.9 (4.0)	6/7	Type I 13	
Ward 2011^30^	Alendronate	109	11.0 (3.6)	47/62	Type I 76, Type III 2， Type IV 11, Unknown 5	2 years
	Placebo	30	11.0 (4.0)	14/16	Type I 37, Type III 3， Type IV 6, Unknown 3	
Bishop 2013^31^	Risedronate	94	8.9 (3.4)	49/45	Type I 60, Type II 16， Type III 2， Type IV 11, Unknown 5	1 year + another 2 years in open‐label
	Placebo	49	8.6 (3.1)	22/27	Type I 29， Type II 8， Type III 3， Type IV 6， Unknown 3	

Type I, Autosomal dominant Fractures with little or no limb deformity, blue sclera, normal stature, hearing loss, dentogenesis imperfecta rare.

Type II Autosomal dominant Lethal perinatal type: undermineralized skull, micromelic bones,“beaded” ribson x‐ray, bone deformity, platyspondyly.

Type III Progressive deformingtype: limb deformities, sclera huevaries, very short stature, dentogenesis imperfect common.

Type IV Sclerae blue, grey, grey/blue, or white, mild/moderate limb deformity with fracture, variable short stature, dentogenesis imperfect common, some hearing loss.

**Table 4:** Study comparison: outcome data reported by individual studies

Study ID	Biochemical markers	BMD	Fracture incidence	Growth	Bone pain	Quality of life
Sakkers 2004 (Olpadronate)	No differences was found in the terms of urinary markers of bone resorption as well as serum concentrations of creatinine, γ‐glutamyl transpeptidase, and aspartate and alanine aminotransferases between the two groups	Unadjusted and adjusted analyses both indicated a greater rise in spinal DXA values with olpadronate than with placebo. Increase spine z score 1.67 SD vs no significant change placebo	Olpadronate treatment was associated with a 31% reduction in relative risk of fracture of long bones (hazard ratio 0·69 [95% CI 0·52–0·91], *P* = 0·01)	No significant change	Not addressed	No significant difference in mobility/ambulation; muscle strength or selfcare
2006 Chevrel (Alendronate)	Decrease in bone resorption markers (collagen peptides, osteocalcin). Alkaline phosphatase unchanged	Increase spine and femur BMD	No differencewas seen in terms of vertebral or peripheral fracture rate between two groups. Not adequately powered	Not addressed	No difference in pain during the study except an increase with alendronate at 36 month	Not addressed
Kok 2007 (Olpadronate)	None	None	None	None	None	Health utility index‐mark III and self‐perception profile for children
Rauch 2009 (Risedronate)	Treatment with risedronate was significantly more effective than treatment with placebo in decreasing serum NTX. No significant treatment difference was observed with regard to changes in the other markers of bone and mineral metabolism: Serum alkaline phosphatase, serum CICP, TRACP5b, urine Ca/creatinine, NTX/creatinine	DXA showed That risedronate Treatment was Associated with a larger increase in lumbar spine BMC and BMD, whereas Changes in lumbar Spine bone projection Area did not differ between groups. No significant difference between The risedronate and placebo groupsWere detected or changes in DXA parameters for hip and total body, as well as for results of pQCT at the radial metaphysic and diaphysis	The Number of fractures per patient ranged from 0 to 2 in the risedronate group and from 0 to 4 in the placebo cohort. None of these outcomes concerning fractures was significantly different between groups	There were also no detectable treatment differences regarding changes in the shape of lumbar vertebral bodie and cortical thickness of the second metacarpal bone. Qualitative evaluation of radiographs did not show any signs of sclerosis in the metaphyses of long bones	The number of patients suffering from bone pain at the end of the study was four in the risedronate group and four in the placebo group. None of these outcomes concerning bone pain was significantly different between groups	Treatment differences in the changes of grip force were not statistically significant
Ward 2011 (Alendronate)	No significant differences between the ALN and placebo groups were observed for changes between baseline and month 24 in serum levels of calcium, phosphorus, creatinine, and urinary calcium to creatinine ratio. The differencein1, 25‐dihydroxy vitamin D reached statistical significance at month 24 mean percent change from baseline at month 24. Twenty‐four months of treatment with ALN was significantly more effective than placebo in decreasing uNTx levels (*P* = 0.001). No significant treatment difference was observed with regard to changes in serum total alkaline phosphatase activity	ALN increased spine areal BMD by 51% *vs* a 12% increase with placebo (*P* = 0.001); the mean spine areal BMD z‐score increased significantly from −4.6 to −3.3 (*P* < 0.001) with ALN, where as the change in the placebo group (from −4.6 to −4.5) was insignificant	The relative risk (95% CI) of having at least one new radiographically confirmed long‐bone fracture between baseline and month 24 was 1.04 for the ALN group, which was not significantly different from1.00. 83% of the ALN patients and 92% of placebo patients sustained at least one investigator‐reported fracture	The mean midline vertebral height was similar between the two groups	Significantly fewer ALN patients experienced bone pain at month 24 than at baseline. The difference between the two groups in terms of the percentage of patients who suffered bone pain was not statistically significant and no significant treatment effect was reported on the number of days per week during which patients experienced bone pain	No statistically significant differences in self‐care or mobility functional skills scaled scores and in grip force was found between the ALN and placebo groups
Bishop 2013 (Risedronate)	Significant mean percentage decreases were noted in urine NTx/creatinine and in serum bone‐specific alkaline phosphatase concentration at 3 and 6 months in the risedronate group. The differences between the risedronate and placebo groups were significant at months 6 and 12 for both markers. Decreases from baseline in either marker during the entire study were greater than 87% in 14 patients. In all but one case, these decreases were in children who were at an age at which reduced bone turnover would be expected because of cessation of longitudinal growth	The mean percentage increase in lumbar spine areal BMD at the end of the placebo‐controlled phase was greater in the risedronate group (16.3%, 95% CI 14.4–18.2) than in the placebo group (7.6%, 5.1–10.1; difference 8·7%, 5.7–11.7; *P* < 0.0001)	Analysis of the time to first clinical fracture during the placebo‐controlled phase showed that risedronate reduced the risk of fractures by 47% (hazard ratio [HR] 0.53, 95% CI 0.31–0.92; log‐rank *P* = 0.0337). Specifically, Kaplan–Meier estimates of the 1‐year fracture rate were 31.4% for the risedronate group and 50.4% for the placebo group	At least one new morphometric vertebral collapse was reported in almost a third of patients in the risedronate group and about a sixth of patients in the placebo group. These fractures were mild in most patients in both treat ment groups. Moderate or severe fractures were noted in similar proportions of patients in the two groups	Not addressed	Not addressed

BMD, bone mineral density.

### *Risk of Bias in Included Studies*

Despite differences in methodological quality, the results of each study were considered equally and were not weighted in the analysis. Further details of the methodological quality of the included studies can be found in Table 2.

Sakkers and Chevrel trials^27^, ^28^ were described as randomized, by computer‐generated random numbers and were deemed adequate. The Chevrel trial was described as double‐blinded (study personnel and participants). For all the trials, an intention‐to‐treat analysis was undertaken. Two participants (one placebo and one treatment) withdrew from the trial but were accounted for in the final analysis. It was also reported that an intention‐to‐treat analysis were performed in the Chevrel trial.

### *Primary Outcomes*

#### *Number of Patients with at Least One Fracture*

Each of the five trials reported on this outcome including three studies in children^27^, ^30^, ^31^ and one trial in adults^28^. The Sakkers trial reported a 31% reduction in relative risk for fracture after treatment with oral olpadronate, and, when analyzed in the review, this produced a hazard ratio of 0.69 (95% CI 0.52 to 0.91) and a significantly decreased fracture number and relative rate (RR) 0.40 (95% CI 0.24 to 0.69)^27^. The Chevrel trial showed that the incidence of vertebral and peripheral fractures was not significantly different between the alendronate and placebo groups. Two vertebral and 17 peripheral fractures occurred in 11 patients in the placebo group versus no vertebral and 17 peripheral fractures in 10 patients in the alendronate group^28^. Similar results are further confirmed by Ward *et al*.^30^. They reported that 83% of the ALN patients and 92% of placebo patients sustained at least one investigator‐reported fracture (*P* = 0.070).

**Figure 2:**
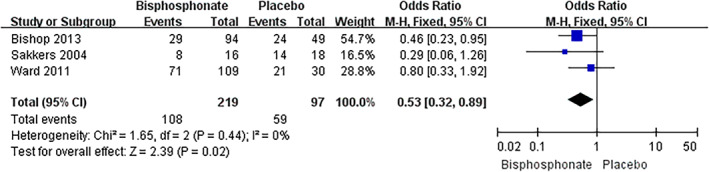
Meta‐analysis of number of patients with at least one fracture comparing oral bisphosphonate with placebo.

Pooled meta‐analysis of three studies^27^, ^30^, ^31^ suggested that there was significant difference between bisphosphonate treated group and placebo group in number of patients with at least one fracture (mean difference 0.53, 95% confidence interval 0.32–0.89, *P* = 0.02) (Fig. 2).

### *Change in Bone Mineral Density (BMD) as Assessed by DEXA*

#### *Mean Percentage Change and Mean Change (Z‐score) in Spine BMD*

Each of the five trials reported on this outcome^27^, ^28^, ^29^, ^30^, ^31^. Analyses of unadjusted and adjusted for baseline group differences in general characteristics both indicated a greater rise in spinal DXA values with olpadronate than with placebo (unadjusted: mean difference 0.046, 95% confidence interval 0.005–0.087, *P* = 0.03; mean difference 0.054, 95% confidence interval 0.012–0.096, *P* = 0.01). Spinal Z‐score increased from –4.98 to –3.31 in olpadronate treated group and from −4.84 to −4.70 in placebo (adjusted group difference 0.74 [95% CI 0.29 to 1.19], *P* = 0.002)^27^. Chevrel *et al*. reported that lumbar spine BMD at 36 months was significantly higher in the alendronate treated group than in the placebo group. The absolute difference between the two groups was 0.058 g/cm^2^ which equals a difference of +9.4 ± 2.0%. The increase was significant higher only in the alendronate group, reaching 10.1 ± 9.8% (an absolute change of 0.061 ± 0.041 g/cm^2^ *P* < 0.001)^28^. A study conducted by Rauch *et al*. showed that DXA showed that risedronate treatment was associated with significantly larger increase in lumbar spine areal BMD during the two years follow‐up. Converted to age‐specific Z‐scores, these results corresponded to a significant mean treatment difference of 0.80 in favor of risedronate^29^. LS areal BMD increased significantly from baseline to month 24 in both ALN and placebo patients, but the mean percentage increase from baseline was 38.8% greater in the ALN group. Converted to age‐specific z‐scores, these results corresponded to a significant mean treatment difference value of 1.18 (95% CI 0.81 to 1.55) in favor of ALN^30^. A recent study finished by Bishop *et al*. reported that at the final follow‐up the mean percentage increase was significantly greater in the risedronate group (16.3%, 95% CI 14.4–18.2) than in the placebo group (7.6%, 5.1–10.1; difference 8.7%, 5.7–11·7; *P* < 0.0001) in lumbar spine areal BMD^31^.

**Figure 3:**
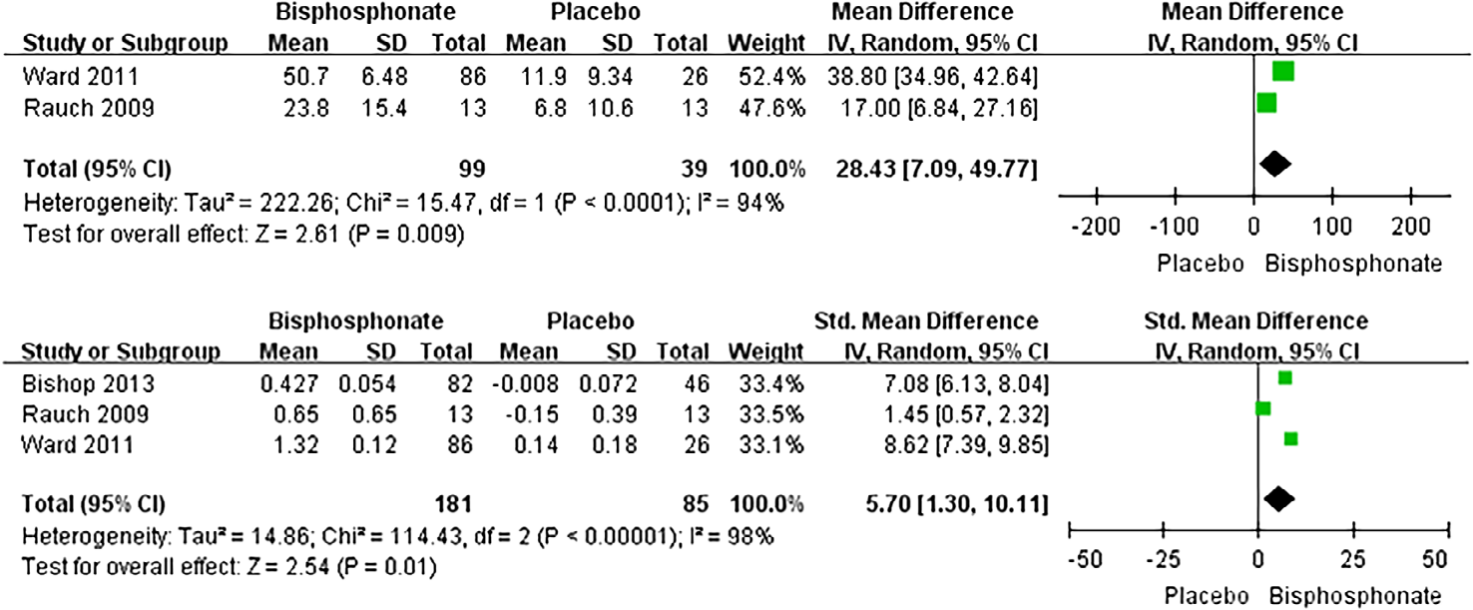
Meta‐analysis of Mean % change and Mean change (Z‐score) in spine BMD comparing oral bisphosphonate with placebo.

Pooled meta‐analysis of two studies^29^, ^30^ suggested that significant difference was noted between bisphosphonate treated group and placebo in mean percentage change in spine BMD (T‐score) (mean difference 28.43, 95% confidence interval 7.09–49.77, *P* = 0.009). The similar effect was shown in the term of mean change (Z‐score) in spine BMD. Meta‐analysis of three studies^29^, ^30^, ^31^ showed that the bisphosphonate treated group presented significantly higher Z‐score compared with placebo group (mean difference 5.70, 95% confidence interval 1.30–10.11, *P* = 0.01) (Fig. 3).

#### *Mean Percentage Change in Total Femur BMD*

Only two trials reported on this outcome^28^, ^29^. Chevrel *et al*. reported that the mean observed change in total femur BMD in the alendronate treated groups and placebo groups was +0.024 ± 0.004 and − 0.002 ± 0.005 g/cm^2^, respectively. This increase in the alendronate group was significantly greater compared with placebo groups (*P* = 0.001). The increase in the alendronate group was significantly greater than that in the placebo group; total femur BMD, MD 3.00 (95% CI 2.73 to 3.27)^28^. However, another study conducted by Rauch *et al*. showed that there was no significant difference between the risedronate treated groups and placebo groups for changes in DXA parameters for hip [Mean (SD), Risedronate: 12.4 (10.8); Placebo: 6.5 (5.9), *P* = 0.11]^29^. As these two trials involve two different populations (children and adults), the meta‐analysis cannot be performed.

### *Secondary Outcomes*

#### *Change in Biochemical Markers of Bone and Mineral Metabolism*

There were five clinical trials that reported on this outcome^27^, ^28^, ^29^, ^30^, ^31^. There were different markers selected for study in the included trials so direct comparison could not be performed. Sakkers *et al*. reported no significant change was found in urine or serum markers (serum concentrations of creatinine,γ‐glutamyl transpeptidase, aspartate and alanine aminotransferases) between the olpadronate and placebo groups^27^. A decrease in bone resorption markers (collagen peptides, osteocalcin) was detected by Chevrel *et al*. in the alendronate‐treated groups, while the levels of alkaline phosphatase remained unchanged^28^. In 2009, Rauch *et al*. also showed that there were no significant differences between the risedronate‐treated and placebo groups in terms of the serum levels of phosphorus, creatinine, 25‐hydroxy vitamin D, 1,25‐dihydroxy vitamin D, PTH, urinary calcium/creatinine ratio, alanine aminotransferase, aspartate aminotransferase, and complete blood count^29^. A significant rise in PTH levels was observed only at month 3 in the ALN treated groups compared with the placebo group (*P* = 0.049). Also statistically significant difference was found in the ALN treated groups in 1, 25‐dihydroxy vitamin D at month 24 compared with the placebo group (*P* = 0.048). uNTx levels decreased significantly in the groups treated with ALN than in the placebo groups (*P* = 0.001). However, there was no significant treatment difference between the two groups with regard to changes in serum total alkaline phosphatase activity^30^. Significant mean percentage decreases were both observed in urine NTx/creatinine and serum bone specific alkaline phosphatase concentration at 3 and 6 months in the risedronate group, while significant difference was noted between the risedronate and placebo groups at months 6 and 12 for both markers^31^.

#### *Bone Histology*

There were two studies^29^, ^30^ that showed histology results. Histomorphometric analysis of these samples by iliac bone biopsies showed that bone formation and resorption parameters, bone size, cortical width, or the amount of trabecular bone were similar between the risedronate and placebo groups^29^. Transiliac histomorphometric safety data showed the ALN group had a significant reduction in osteoid volume per bone volume after treatment. This difference between treatment groups was highly significant in the semiquantitative evaluation of iliac bone samples which were obtained at the end of the treatment period revealing the presence of at least one large osteoclast (>50 mm). However, no significant treatment difference was found between the two groups regarding the presence of calcified cartilage^30^.

### *Growth*

Three trials reported on this outcome^27^, ^29^, ^30^. Sakkers *et al*. reported no difference was shown in seated height or radiographic assessments of lumbar vertebral height between olpadronate treated groups and placebo at final follow‐up^27^. Rauch *et al*. also demonstrated that no detectable treatment differences were observed regarding changes in the shape of lumbar vertebral bodies and cortical thickness of the second metacarpal bone^29^. The mean midline vertebral height was also similar between the ALN and placebo groups (*P* = 0.444)^30^.

### *Bone Pain*

Three trials reported bone pain change^28^, ^29^, ^30^. Chevrel *et al*. reported that the pain score was similar in the two groups from 0 to 30 months and reported end‐of‐trial data that showed an increase at final follow‐up with alendronate treated groups (mean 1.30, 95% CI 0.14 to 2.46)^28^. Also, another study confirmed the results that there was no significant difference between these two groups^29^. However, one trial completed by Ward *et al*. reported that significantly fewer ALN patients experienced bone pain at month 24 than at baseline (*P* = 0.001). But there was no significant difference between the ALN and placebo groups in the percentage of patients who experienced bone pain (*P* = 0.065)^30^. As these two trials involving different populations (children and adults), the meta‐analysis cannot be performed.

### *Quality of Life*

Five included trials evaluated at least one quality of life outcome^22^, ^27^, ^28^, ^29^, ^30^. Sakkers reported that no changes were found in the changes of grip or hip flexor strength or mobility when comparing olpadronate to placebo, and the difference in changes was found to be similar between the two groups^27^. Hearing was assessed in the study by Chevrel *et al*., which found no difference between the alendronate‐treated group and placebo group (mean: ‐0.10, 95% CI ‐2.88 to 2.68)^28^. No statistically significant difference was observed by two studies in self‐care or mobility functional skills scaled scores and in grip force^29^, ^30^. We found only slight differences in quality of life, which was measured using self‐perception profile for children (SPPC) and health‐utility index (HUI) in favor of the bisphosphonate group. A small but not significant decrease in pain was detected in the bisphosphonate group^22^.

## Discussion

Six randomized controlled studies were included in our systematic and meta‐analysis^22^, ^27^, ^28^, ^29^, ^30^, ^31^. Two studies conducted by Sakkers *et al*. and Kok *et al*.^22^, ^27^ reported on the same patient groups with olpadronate but involving different results: the former focuses on skeletal effects and functional outcomes while the latter one focuses on quality of life. Five trials are investigating the effect of oral bisphosphonate clinical results in children with osteogenesis imperfecta while only one trial studies the effect of oral bisphosphonate in adults with osteogenesis imperfecta^28^. Five studies enrolled children^22^, ^27^, ^29^, ^30^, ^31^ and one enrolled adults^28^.

All the included studies investigated changes to varying degrees in BMD, fracture rate, markers of bone turnover, growth, pain, and quality of life with bisphosphonate therapy. For a summary of outcomes reported by different trials, see Table 4. All studies assessing BMD independently reported significant increases after treatment with either oral or IV bisphosphonate and at separate sites (spine, hip, femur)^27^, ^28^, ^29^, ^30^, ^31^. However, it is difficult to compare all these trials directly as different populations were included (adults versus children; for accurate comparisons children cannot be compared to adults due to high bone turnover during childhood and adolescence and open epiphyses). Additionally, different reporting indices were used (Z‐score versus total BMD). But data from some of these studies can contribute to the analysis when the same population and reporting indices were employed as well as the same locations were measured. Pooled meta‐analysis of two studies^29^, ^30^ suggested that significant difference was noted between bisphosphonate treated group and placebo in mean percentage change in spine BMD (mean difference 28.43, 95% confidence interval 7.09–49.77, *P* = 0.009). A similar effect was shown in the term of mean change (Z‐score) in spine BMD by three studies^29^, ^31^, ^32^. Both randomized^29^, ^31^, ^32^ and non‐randomized^33^ studies all reported that oral bisphosphonate therapy has shown increases in BMD. The majority of the patients enrolled by Bishop *et al*. and Sakkers *et al*. were children with mild forms of the disease. They concluded that the oral bisphosphonate treatment is not suited to severe types of OI, but is suited to mild types. The effect of oral bisphosphonate in increasing and reducing fracture rates in BMD are consistent with those treated by intravenous bisphosphonate in uncontrolled, observational studies^12^, ^14^ and randomized controlled trial^19^. Each of the four trials reported on this outcome including three studies on children^27^, ^30^, ^31^ and one trial on adults^28^. As mentioned before, an increase in the growth and BMD in children and adolescents with OI, together with the trend for decreased fractures, makes data comparison from all the trials extremely difficulty. The meta‐analysis was performed when the same indices were reported among the studies. Pooled meta‐analysis of three studies^27^, ^30^, ^31^ suggested that there was significant difference between bisphosphonate treated group and placebo group in the number of patients with at least one fracture (mean difference 0.53, 95% confidence interval 0.32–0.89, *P* = 0.02).

Improvement in the height of vertebral bodies and cortical width has been shown among the children with OI treated with IV bisphosphonate^19^, ^35^, ^36^, ^37^, ^38^. Similar increases were observed by DiMeglio *et al*. in terms of the height of vertebral bodies between high‐dose oral bisphosphonate and IV treatment^39^. In contrast, the oral bisphosphonate (alendronate, risedronate and olpadronate) therapy was successful in increasing the lumbar BMD but was not accompanied by improvement in other skeletal parts such as metacarpal and iliac cortical width^27^, ^29^, ^30^, ^32^. One explanation for this phenomenon is that primary and secondary spongiosa are the main component of the vertebral body. More and more primary spongiosa are converted to secondary spongiosa during the child growing phase^34^.

Mild and hardly noticeable change in the bone metabolism has been reported among the patients with OI treated with oral bisphosphonate even though the definite treatment effect on lumbar spine areal BMD has been shown^27^, ^28^, ^29^, ^30^, ^32^. Sakkers *et al*. reported that no significant change was found in urine or serum markers (serum concentrations of creatinine,γ‐glutamyl transpeptidase, aspartate and alanine aminotransferases) between the olpadronate and placebo groups^27^. Rauch *et al*. also showed that there was no significant differences between the risedronate‐treated and placebo groups in terms of the serum levels of phosphorus, creatinine, 25‐hydroxy vitamin D, 1,25‐dihydroxy vitamin D, PTH, urinary calcium/creatinine ratio, alanine aminotransferase, aspartate aminotransferase, and complete blood count^29^. No commonly accepted biochemical markers of bone turnover are used, but it is a public assumption that they can act as a proxy monitoring the efficacy of therapy. The varied markers selected for study in the trials made the direct comparison impossible. These different biological bone turnover markers can help doctors to assess the drug therapeutic effect of the bisphosphonates, drug dosing, and participant compliance.

Bone pain and mobility are the two main key clinical indices that have direct influence on the quality of life of the participants. Improvements in terms of these parameters have been shown in several studies^19^, ^34^, ^40^, ^41^. However, no obvious change in mobility was noted after 2 years of oral bisphosphonates^27^, ^30^ and no improved to bone pain was detected in the trial by Bishop *et al*. using oral risedronate^32^. One of the possible reasons for this phenomenon is that oral bisphosphonates provided less benefit than IV treatment due to its lower therapeutic effect by oral administration. However, one trial conducted by Chevrel *et al*. found no difference in self‐reported pain scores, with the exception of increased pain with bisphosphonates at 36 months. They also assessed the hearing and found no change in Rinne testing^28^. Taken together, no consistent improvements in these quality‐of‐life indicators with bisphosphonate administration was demonstrated by the current available literature.

Importantly, oral bisphosphonate administration was associated with few adverse effects. Ward *et al*. reported that mean bisphosphonate oral bioavailability is similar among the child and adult participants and the individual oral bioavailability varies as much as ten‐fold^42^. Chevrel *et al*. reported that gastrointestinal symptoms were more common in the group of patients treated with alendronate than in those placebo ones, although these symptoms were not responsible for treatment withdrawal. Their results are in contrast with these other four studies^27^, ^29^, ^30^, ^31^, which indicated that gastrointestinal symptoms were not more common in ALN groups than in placebo groups. Based on current evidence, oral bisphosphonate administration treated for 1–3 years appears to be safe and well tolerated in the patients affected with OI. Most of the adverse effects reported in the trials are few and minor including gastrointestinal complaints, fever, headache, nausea, arthralgia, and others, and the drugs are generally well tolerated.

### *Conclusion*

Significant improvement in lumbar areal BMD in patients affected with OI have been shown when treated with oral bisphosphonates, even though only a small population was enrolled. We cannot draw a definite conclusion that the increase in BMD can translate into fracture reduction and clinical functional improvement. The optimal method, dose, type, initiation and duration of oral bisphosphonates therapy still remains unclear. Well designed, adequately‐powered, placebo‐controlled RCTs investigating the effects of oral bisphosphonates on fracture reduction are important to determine improvements to quality of life in both children and adults.
